# Hot Crack Formation Mechanism and Inhibition of a Novel Cobalt-Based Alloy Coating during Laser Cladding

**DOI:** 10.3390/ma17163914

**Published:** 2024-08-07

**Authors:** Pengfei Yang, Nannan Lu, Jingjing Liang, Yimo Guo, Guangrui Zhang, Xiu Song, Yizhou Zhou, Xiaofeng Sun, Jinguo Li

**Affiliations:** 1 Key Laboratory for Anisotropy and Texture of Materials (Ministry of Education), School of Materials Science and Engineering, Northeastern University, Shenyang 110819, China; pfyang0721@163.com (P.Y.); songxiu@mail.neu.edu.cn (X.S.); 2 Shi-Changxu Innovation Center for Advanced Materials, Institute of Metal Research, Chinese Academy of Sciences, Shenyang 110016, China; jjliang@imr.ac.cn (J.L.); ymguo24h@imr.ac.cn (Y.G.); grzhang@imr.ac.cn (G.Z.); yzzhou@imr.ac.cn (Y.Z.); xfsun@imr.ac.cn (X.S.)

**Keywords:** novel cobalt-based alloy coating, laser cladding, process optimization, hot cracking mechanism, crack inhibition

## Abstract

Laser cladding provides advanced surface treatment capabilities for enhancing the properties of components. However, its effectiveness is often challenged by the formation of hot cracks during the cladding process. This study focuses on the formation mechanism and inhibition of hot cracks in a novel cobalt-based alloy (K688) coating applied to 304LN stainless steel via laser cladding. The results indicate that hot crack formation is influenced by liquid film stability, the stress concentration, and precipitation phases. Most hot cracks were found at 25°–45° high-angle grain boundaries (HAGBs) due to the high energy of these grain boundaries, which stabilize the liquid film. A flat-top beam, compared to a Gaussian beam, creates a melt pool with a lower temperature gradient and more mitigatory fluid flow, reducing thermal stresses within the coating and the fraction of crack-sensitive, high-angle grain boundaries (S-HAGBs). Finally, crack formation was significantly inhibited by utilizing a flat-top laser beam to optimize the process parameters. These findings provide a technical foundation for achieving high-quality laser cladding of dissimilar materials, offering insights into optimizing process parameters to prevent hot crack formation.

## 1. Introduction

Austenitic stainless steel is commonly utilized in the nuclear power industry due to its exceptional corrosion resistance, excellent machinability, and relatively low cost. It finds application in manufacturing reactor bearings, hook claws, and other crucial transmission components within the reactor. However, prolonged exposure to extreme conditions, such as high temperature, high pressure, and severe wear, can lead to surface damage and failure [1,2]. It is customary to employ surface treatment technologies to enhance the surface properties of these components to extend their service life.

As a novel surface treatment technology, laser cladding (LC) offers several advantages over other surface strengthening techniques: (1) minimal coating dilution, reduced heat-affected zone, limited deformation, fine grain size, and compact structure; (2) a wide range of materials can be utilized as cladding material including self-melting powder, ceramic powder, and composite powders; (3) high automation level, ensuring environmental friendliness and material conservation; (4) small melt pool size, flexible powder feeding mechanism, and uniform powder heating process, leading to enhanced crack resistance. Consequently, it has gained widespread application in coating preparation and additive manufacturing of large components [3,4,5,6].

Cobalt-based high-temperature alloys exhibit superior high-temperature oxidation resistance, corrosion resistance, and hardness compared to iron-based and nickel-based alloys [7,8]. As a result, cobalt-based alloys are frequently employed as cladding materials for stainless steel components. However, the significant disparities in physical and chemical characteristics between stainless steel and cobalt-based alloys can lead to element diffusion at heterogeneous interfaces, thereby impacting the coating’s phase composition and printability. Consequently, achieving crack-free cladding of cobalt-based alloy coatings through laser cladding remains a critical technical challenge that requires urgent resolution [9].

Currently, numerous researchers have conducted extensive research on the issue of laser-cladding-induced cracking in cobalt-based alloy coatings, with a primary focus on process optimization, strategic approaches, and alloy composition enhancement. Zhang et al. [10] employed the hot wire laser cladding technique to deposit cobalt-based alloy coatings onto an A36 substrate. The findings revealed that an increase in wire feed rate led to an expansion in both the width and length of the melt pool while decreasing the cooling rate; it also resulted in increased secondary dendrite spacing and an enhanced wetting angle of the coating. Conversely, the opposite trends were observed when the scanning speed increased (except for the constant dilution ratio). These results suggested the importance of achieving optimal matching between these two speeds for successful formation processes. Ilanlou et al. [11] proposed a predictive model based on three main process parameters (laser power, scanning speed, and powder feeding rate) to prevent the formation of defects when Co-Cr-W coatings are deposited on martensite stainless steel surfaces. Experimental investigations demonstrated that the optimal coating could be achieved within a 10–20% dilution ratio range, with a wetting angle below 45°, and when the width-to-height ratio for the melt pool ranging from 1/4 to 1/2. The deposition of Stellite 21 alloy multi-layers on an Inconel 718 substrate by Smoqi et al. [12] demonstrated a reduced tendency to crack when the coating was applied onto a preheated substrate using a medium energy density. Furthermore, cracking of the coating at a higher energy density could be attributed to the segregation of Cr and Mo elements, which form hard and brittle phases in the inter-dendritic regions. These phases hindered liquid flow, resulting in insufficient replenishment and ultimately leading to cracking under residual thermal stresses.

The latest research has demonstrated that, in comparison to the conventional cobalt-based alloy, a novel cobalt-based alloy exhibits exceptional corrosion and creep resistance properties while maintaining its hardness [13]. Meanwhile, the temperature-bearing capacity of the material is significantly improved [14]. This provides another new option for cladding materials in the nuclear power industry. However, previous studies have primarily focused on traditional cobalt-based alloy specimens and lack investigations into novel cobalt-based alloy coatings. Therefore, it is necessary to study the cracking mechanism of novel cobalt-based alloys to obtain novel crack-free, cobalt-based alloy coatings.

The present study focuses on the preparation of a novel cobalt-based alloy coating (K688) on a 304LN substrate using laser cladding. A meticulous analysis was conducted to investigate the influence of various process parameters on the microstructure and geometrical morphology of the single-track deposition zone. Furthermore, the phase composition of the coatings and the underlying mechanisms of crack formation were subjected to extensive investigation. By optimizing the process parameters and adjusting the laser beam pattern, successful crack inhibition cladding was accomplished, providing valuable insights for further explorative research in the field.

## 2. Materials and Methods

### 2.1. Experimental Materials

**Figure 1:**
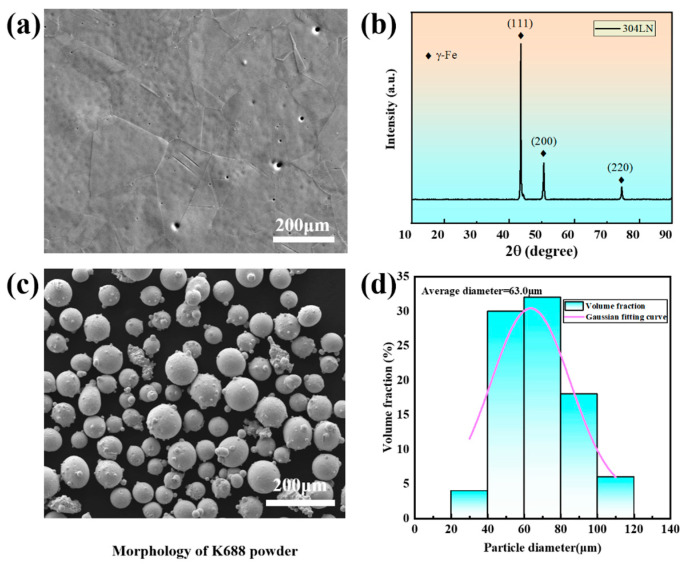
(**a**,**b**) Microstructure of 304LN stainless steel; (**c**) morphology of K688 powder; (**d**) particle diameter distribution of powder.

In this study, 304LN was utilized as the substrate material (50 mm × 20 mm × 10 mm). Before the cladding, the surface was ground using sandpaper to eliminate rust and oxide film, followed by cleaning with anhydrous ethanol, to facilitate subsequent cladding. The 304LN was composed of 0.01C, 0.11N, 0.52Si, 1.49Mn, 8.72Ni, 18.26Cr, and 70.89Fe (wt%). Analysis through the X-ray diffractometer (XRD) pattern and secondary electron image (refer to Figure 1a,b) revealed a microstructure of single austenite, with a few twins present within the austenite crystal structure.

The K688 alloy powder used in the cladding experiments was provided by the Institute of Metal Research, Chinese Academy of Sciences. Figure 1c shows the micromorphology of the powder observed by a scanning electron microscope (SEM), with Image Pro Plus employed in a statistical analysis of its particle size, as depicted in Figure 1d. The particle diameter distribution ranges from 40 µm to 100 µm, with an average particle diameter of approximately 63.0 µm. Before the cladding process, the powder was subjected to vacuum drying treatment (1 h) within a drying box to prevent air hole formation resulting from uneven powder feed and water evaporation. The chemical composition of K688 powder is shown in Table 1.

### 2.2. Cladding Equipment and Process

**Figure 2:**
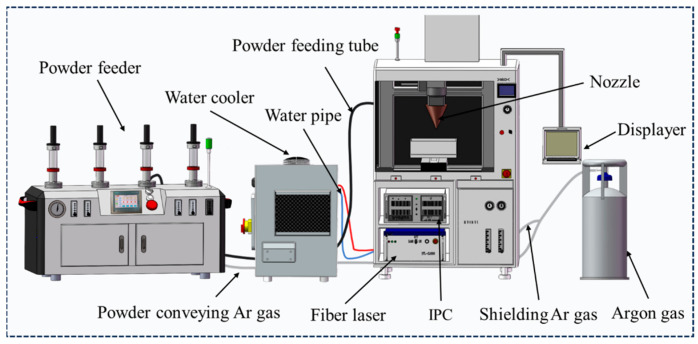
Laser cladding equipment.

The experiments were carried out using laser-directed energy deposition equipment of the Institute of Metal Research, Chinese Academy of Sciences. The equipment (Metal+30E-200, HUIRUI, Nanjing China), as shown in Figure 2, consists of a fiber laser, a three-axis Computerized Numerical Control (CNC), an Industrial Personal Computer (IPC), a water cooler, a powder feeder, and a coaxial annular powder feeding nozzle. The laser has a maximum output power of 2000 W with a Gaussian beam and a spot diameter of 2 mm. A protective gas flow rate of 6 L/min is maintained, with a distance of 15mm between the substrate and nozzle to prevent the oxidation of the coating. Additionally, a semiconductor fiber laser with a maximum output power of 4000 W was also utilized in this study (RC-LDM-4030, RAYCHAM LASER, Nanjing, China). This particular laser operates with a flat-top beam pattern and a spot diameter of 2.5 mm. Similar to the previous setup, experiments were conducted in an Argon environment, with a protective gas flow rate of 6 L/min, and a 10 mm distance between the nozzle and substrate.

The single-track cladding process was optimized through a one-factor experiment, with the specific process parameters presented in Table 2. Following this, a sample size of 40 mm × 10 mm × 0.5 mm was fabricated using multi-track cladding to create a single layer, employing three sets of parameters with overlap ratios of 20%, 31%, and 41%. This aimed to investigate the impact of the overlap ratio on coating quality, crack density, dilution ratio, etc. Finally, a set of optimal process parameters was obtained through multi-objective optimization to prepare a virtually crack-free coating.

### 2.3. Characterization Methods

**Figure 3:**
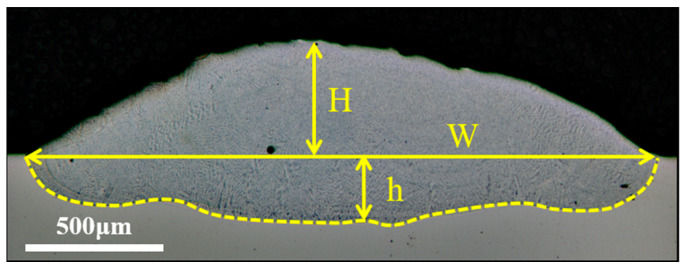
Geometric dimensions of the single-track deposition region.

As shown in Figure 3, the geometric dimensions of the single-track deposition region mainly include W (deposition width), H (deposition height), and h (remelting depth), which are obtained by using Photoshop’s measurement tool. The geometric dilution ratio (D) was defined as [15]

D = h/(H + h)(1)

where h is the remelting depth; and H is the deposition height. The crack density (η) was defined as the proportion of cracks per unit area [16]:

η = a/A(2)

where a is the crack area; and A is the cross-sectional total area. To ensure precise statistical results, the analysis of crack density was conducted using Image Pro Plus software. Four optical microscope (OM) images were consecutively captured under equal magnification, and the final result was determined by considering the average value.

The cladding specimens were cut into metallographic samples (10 mm × 10 mm × 4 mm) by wire cutting. Initially, the surface of the samples was ground sequentially with 180~2000# sandpaper and then mechanically polished with 2.5 μm diamond grinding paste. Subsequently, the samples were cleaned with alcohol and dried using a hair dryer. Finally, the metallographic samples were electrochemically etched to observe the structure and morphology. We used an electrochemical etching voltage of 10 V and an etching time of 12 s. The etchant composition was 42 mL H_3_PO_4_ + 34 mL H_2_SO_4_ + 24 mL H_2_O. The OM (DM4M, Leica, Wetzlar, Germany) was used to observe the structure and morphology. A field emission scanning electron microscope (Sigma 300, ZEISS, Jena, Germany) equipped with an energy-dispersive X-ray spectrometer (EDS) (Xplore 30, Oxford, UK) was employed for microstructural observation and compositional analysis. The dendritic core regions, inter-dendritic regions, cracks, and peripheral structures were characterized using secondary electron (SE) and back-scattered electron (BSE) images. Crystal orientation information was obtained via Electron Back-Scattering Diffraction (EBSD) (Symmetry S2, Oxford, UK) at an acceleration voltage of 20 kV, working distance of 8.2 mm, and detection step length of 0.4 μm. Before the experiment, apart from conventional treatments such as grinding and polishing, the samples used for EBSD analysis also required Argon ion etching (PECS ΙΙ685, GATAN, Pleasanton, CA, USA) at a working voltage of 3 kV for 1 h. The phase composition of the coatings was identified by using an X-ray diffractometer (XRD) (Smart Lab, Rigaku, Osaka, Japan). XRD is performed in the test range of 20–90° (2θ) with a tube voltage of 40 kV, tube current of 15 mA, scanning speed of 20°/min, and test step length of 0.02° (Δ2θ), and Cu Kα is used as the radiation source. Before the experiment, a polished coating surface section was selected for XRD testing and analysis; the coating surface section is also referred to as the XY plane below.

## 3. Results

### 3.1. Morphology and Geometric Dimensions of Single-Track Deposition Region with Different Process Parameters

**Figure 4:**
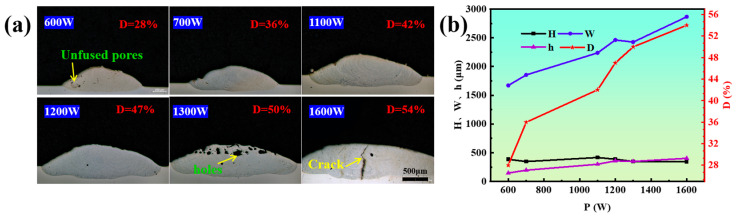
(**a**) Morphologies of single-track coatings at different laser powers (10 mm/s, 6 g/min); (**b**) effects of laser power on the dimensions and dilution ratio of single-track coatings (10 mm/s, 6 g/min).

The morphology plays a crucial role in evaluating the quality of coatings. Research on the quality of single-track cladding can provide a fundamental basis for subsequent multi-track cladding [17,18]. Figure 4a shows the morphology of the coating cross-section under different laser powers. The results demonstrate that the sample mainly consists of a cladding zone and substrate zone, and a good metallurgical bonding can be achieved between the coating and the substrate. In addition, when the laser power is 1300 W, there are lots of holes at the top region of the coating. However, if the power is increased to 1600 W, the single-track coating will generate cracks due to the thermal stress being too large at this time [19]. To further elucidate how laser power affects geometric dimensions and the geometric dilution ratio in single-track coatings, measurements were taken from OM photographs, shown in Figure 4a and presented graphically in Figure 4b. It was found that when the laser power was increased from 600 W to 1100 W, the increasing laser heat input increased the amount of melted powder and substrate. Thus, the depth, width, height, and geometric dilution ratio increased, while the height of the coatings decreased slightly after 1100 W. This is because with the increase in laser power, on the one hand, the amount of molten powder increases, resulting in an increase in height, but the increase is limited. On the other hand, the increase in molten pool fluidity results in a decrease in height. Therefore, for the above two aspects, at this time, the height reduction caused by the increase in molten pool fluidity plays a dominant role. Therefore, the height of the coating tends to decrease [20]. Additionally, the research reveals that laser power primarily influences the width of the coatings and the geometric dilution ratio.

**Figure 5:**
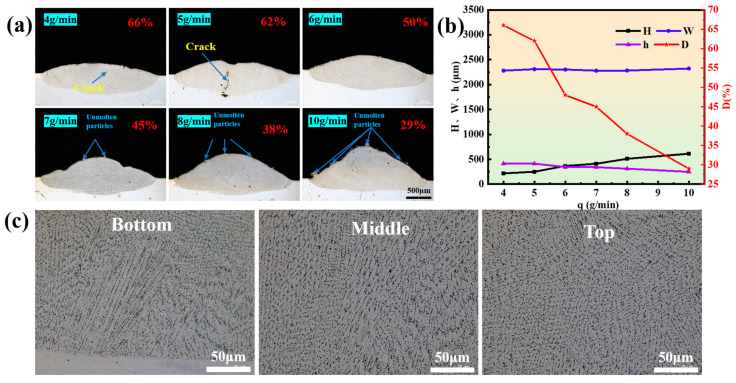
(**a**) Morphologies of single-track coatings at different powder feeding rates (1100 W, 10 mm/s); (**b**) effect of powder feed rates on the dimensions and dilution ratio of the coatings (1100 W, 10 mm/s); (**c**) microstructures of the bottom, middle, and top of single-track coatings (1100 W, 10 mm/s, 7 g/min).

Figure 5a shows the morphologies of the coating cross-section at different powder feeding rates. It can be found that there are obvious cracks in the middle region of the coating when the powder feeding rate is 4 g/min or 5 g/min. On the contrary, when the powder feeding rate is increased to 6 g/min, the cracks will disappear. However, when the powder feeding rate is increased to 10 g/min, unmelted powder particles appear on the surface of the coating. Therefore, to avoid cracks and unmelted powder particles in the coating, a higher powder feeding rate should be selected for laser cladding [21]. Figure 5b reveals the effect of the powder feeding rate on the coating’s geometric dimensions and geometric dilution ratio, from which it can be seen that the feeding rate mainly affects the coating height and geometric dilution ratio, which is shown as follows: with the increase in the feeding rate, the coating height increases slowly and the geometric dilution ratio decreases sharply. Figure 5c shows the microstructure of the coating’s bottom, middle, and top areas. The bottom exhibits planar crystal growth attributed to a larger G (temperature gradient)/R (solidification rate) [22]. Moving inward from the solidifying pool, G/R decreases, leading growth to shift from planar to columnar crystals. At the top, characterized by low G/R and notable compositional supercooling, fine dendritic and equiaxed crystals mainly form [23].

**Figure 6:**
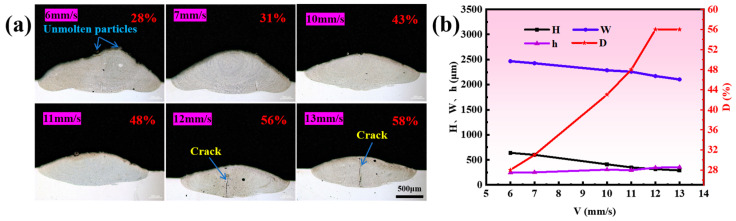
(**a**) Morphologies of single-track coatings at different scanning speeds (1100 W, 6 g/min); (**b**) effects of scanning speed on the dimensions and dilution ratio of single-track coatings (1100 W, 6 g/min).

As illustrated in Figure 6a, the risk of coating cracking increases significantly with an increasing scanning speed. This is due to an increase in the solidification rate of the molten pool with the increase in scanning speed, causing an increase in G, which in turn leads to an increase in the tensile stress in the coating, and an increase in the cracking ratio [24,25]. Furthermore, the results in Figure 6b demonstrate that the scanning speed significantly influences the geometric dilution ratio, followed by the coating’s height, width, and depth. Specifically, the scanning speed is positively correlated with the geometric dilution ratio and the depth, while it is negatively correlated with the width and the height. These changes were attributed to a reduction in the laser heat input per unit of time and the amount of powder fed into the melt pool per unit.

Based on the impacts of the three process parameters on cracks and the geometric dilution ratio, the single-track coating process window was optimized: a low to medium power (700–1200 W), low to medium scanning speed (6–10 mm/s), and high powder feeding rate (6–8 g/min) were chosen for the subsequent cladding.

### 3.2. The Behavior of Cracks and the Diffusion of Elements at Different Overlap Ratios

**Figure 7:**
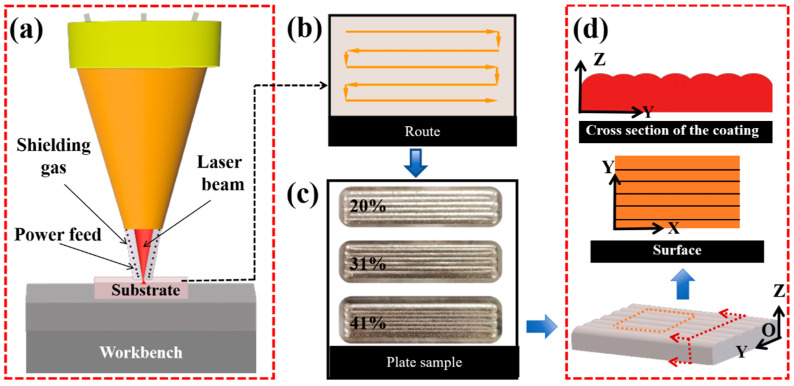
(**a**) Schematic diagram of the laser cladding process; (**b**) deposition strategy; (**c**) the macroscopic morphology of the coatings (1100 W, 8 mm/s, 6 g/min); (**d**) the XY and YZ planes sectioned for the specimen.

Coatings with different overlap ratios were prepared using a laser power of 1100 W, a scanning speed of 8 mm/s, and a powder feeding rate of 6 g/min. Figure 7a depicts a schematic diagram of the complete cladding process. The experiment was carried out using a serpentine reciprocating sweep strategy (Figure 7b). The macroscopic morphology of the coatings is shown in Figure 7c, and the surfaces of all the samples exhibited a bright metallic luster, no macroscopic cracks, and unmelted powders. The coating was sectioned along the planes parallel to XY and YZ, respectively, as depicted in Figure 7d, to examine the presence of microcracks within it.

**Figure 8:**
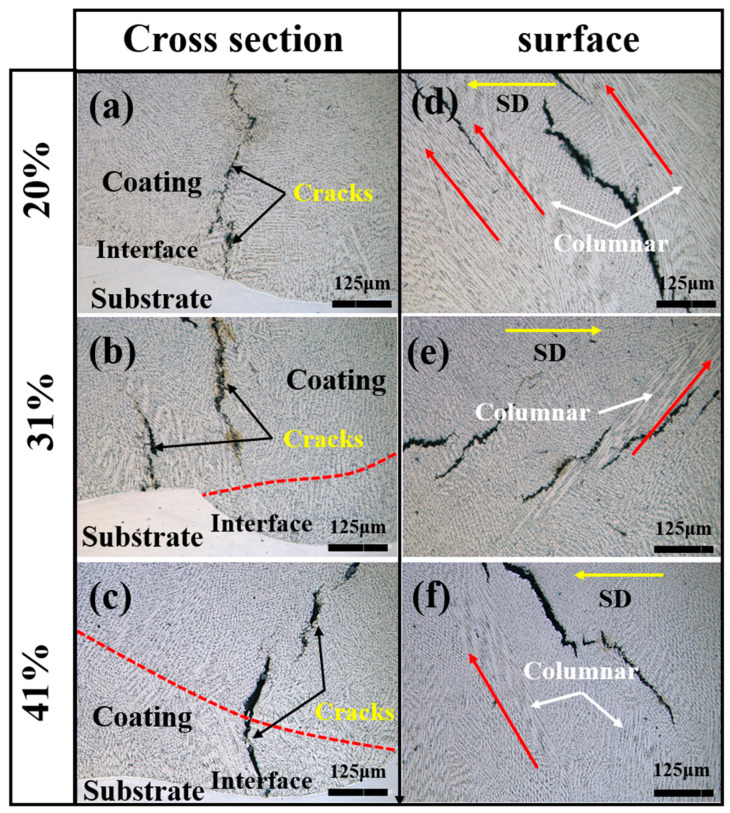
Microstructures of the coatings with different overlap ratios (1100 W, 8 mm/s, 6 g/min): (**a**–**c**) XZ plane; (**d**–**f**) XY plane.

Figure 8 illustrates the microstructures of cladding coatings with varying overlap ratios. The red dashed line demarcates the boundary of the melt pool, the red arrow signifies the direction of dendrite growth, and the yellow arrow denotes the direction of laser scanning. From the cross-sectional structures in Figure 8a–c, it can be observed that microcracks were present within the coating under all three overlap ratios. The cracks originated at the fusion interface of the samples and rapidly expanded in the Z direction after initiation. Some cracks terminated in the equiaxed crystalline region, while others crossed the equiaxed crystalline region and expanded to the coating surface. Figure 8d–f illustrate the distribution of cracks in the XY plane. The cracks at the top exhibit an oblique orientation of 45° with respect to the scanning speed direction (SD), rather than being primarily transverse or longitudinal. Furthermore, the majority of these cracks are predominantly distributed along the grain boundaries of both columnar and equiaxed crystals.

**Figure 9:**
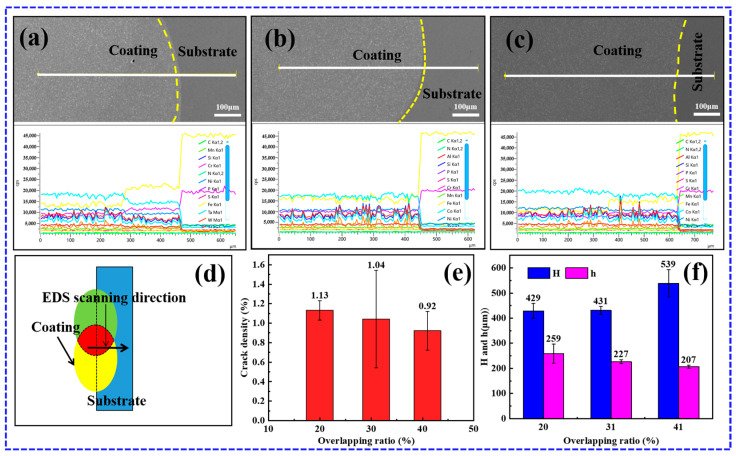
(**a**–**c**) EDS line scan results from the YZ plane of the coatings at 20%, 31%, and 41% overlap ratios (1100 W, 8 mm/s, 6 g/min); (**d**) diagram of EDS line scanning position; (**e**) effect of overlap ratio on coating height and remelting depth (1100 W, 8 mm/s, 6 g/min); (**f**) effect of overlap ratio on crack density (1100 W, 8 mm/s, 6 g/min).

Figure 9a–c presents the EDS line scan results from the cladding to the substrate. It can be observed that there is a significant decrease in the content of Co and Ni elements as the transition progresses from the cladding coating to the substrate. In contrast, there is a notable increase in the Fe and Cr elements. The mutant layer is discernible at the fusion interface, exhibiting a thickness of approximately 10 µm. As the overlap ratio increases from 21% to 40%, the dilution of Fe and Cr to the coating is slowly reduced. However, it is important to note that although a large overlap ratio can reduce the dilution of Fe and Cr elements in the coating, it cannot be avoided entirely. Figure 9d presents a schematic diagram of the EDS scanning position. Figure 9e shows that the geometric dilution ratio of the coating decreases with the increase in the overlap ratio, which is consistent with the results of the EDS line scanning. Furthermore, the measured statistics of the crack density under different overlap ratios, as shown in Figure 9f, indicate that the overlap ratio is negatively correlated with the crack density, suggesting that increasing the overlap ratio is beneficial for inhibiting coating cracking.

### 3.3. Phase Composition of the Coatings

**Figure 10:**
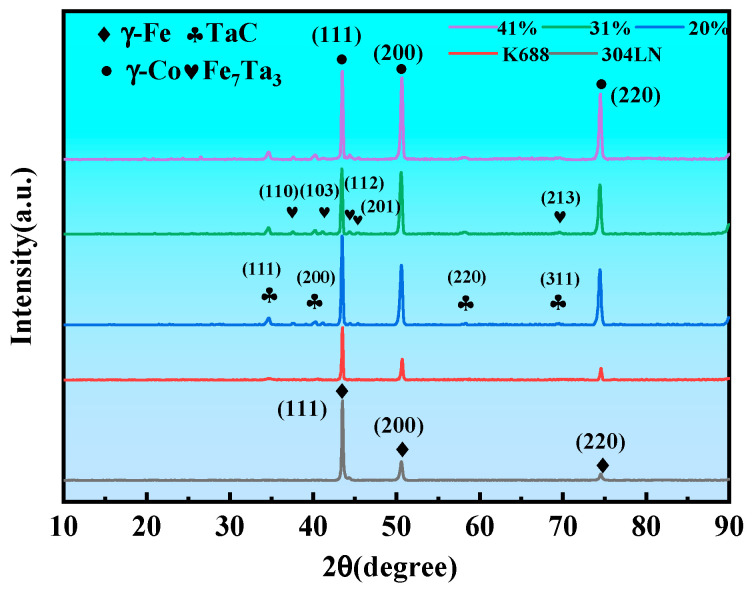
XRD patterns of the powder, substrate, and coatings (1100 W, 8 mm/s, 6 g/min).

To elucidate the underlying cause of coating cracking and subsequently inhibit this detrimental process, it is imperative to determine the phase composition of the coatings accurately. Cross-sections of the samples were examined by XRD, and the results are shown in Figure 10. This figure illustrates that the K688 powder primarily comprises γ-Co and MC-type carbides (TaC). Additionally, the figure reveals the emergence of Fe_7_Ta_3_ characteristic diffraction peaks in the coatings with varying overlap ratios [26], in addition to the γ-Co and MC-type carbides (TaC). This evidence suggests that the diffusion of the Fe element from the substrate resulted in the formation of a new phase in the coatings.

**Figure 11:**
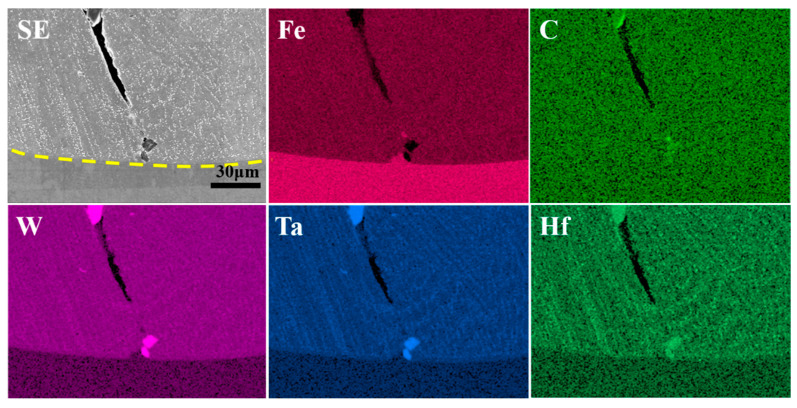
Plane distribution of elements in the coating (1100 W, 8 mm/s, 6 g/min, 41%).

Figure 11 demonstrates the distribution of elements within the coating. It can be observed that the K688 coating is comprised of a matrix and precipitated phases. The precipitated phases are predominantly precipitated within the inter-dendritic regions. Additionally, it can be noted that the elements W, Ta, and Hf are primarily concentrated in the inter-dendritic regions, while Fe is concentrated in the dendritic core regions. This phenomenon can be attributed to the fact that LC is a non-equilibrium solidification process in which the redistribution of solute atoms along the solid–liquid interfacial front leads to the segregation of elements between the inter-dendritic regions [27].

**Figure 12:**
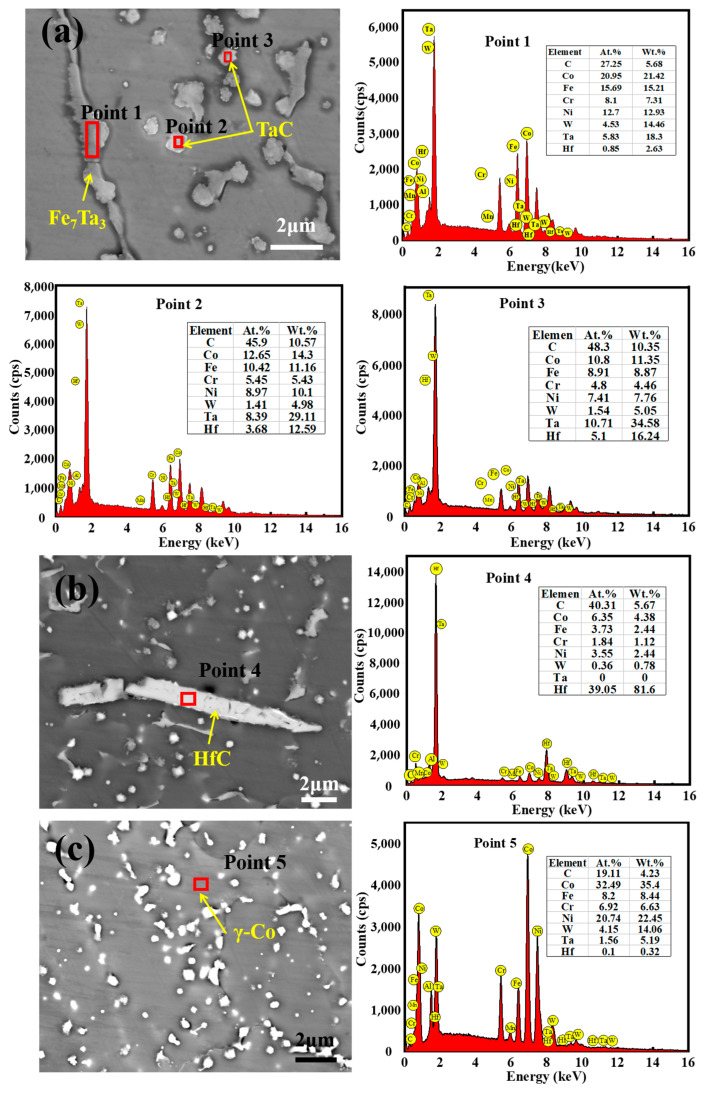
BSE images and EDS analysis of the coating (1100 W, 8 mm/s, 6 g/min, 41%): (**a**) bottom; (**b**) middle; (**c**) top.

Figure 12a–c provides BSE images of the coating cross-section at the bottom, middle, and top, respectively. It can be observed that the precipitated phases in the coatings exhibit various structural morphologies, including point, bone frame, and strip shapes. In Figure 12a, the precipitated phase in the form of darker strips (point 1) is primarily composed of Fe and Ta elements. Quantitative analysis by EDS indicates that the atomic ratio of Fe to Ta is approximately 7:3. Combining this result with the findings of the XRD analysis in Figure 10, it can be concluded that the corresponding phase is Fe_7_Ta_3_. Furthermore, bright white point-like precipitated phases can be observed in the figure. Based on the results of XRD on display, and combined with the EDS analysis results, we can surmise that these phases are TaC. Additionally, it was found that these carbides can also be distributed in a bone-like manner, which is similar to the findings of Berthod and Chen et al. [28,29]. As illustrated in Figure 12b, there are also thick and long strips of HfC in the middle region of the coating, which XRD did not detect. This is likely because the amount of HfC present is relatively low. A BSE image of the top of the coating is shown in Figure 12c. The top of the coating also contains a large amount of point-like TaC. In conjunction with Figure 12a–c, the amount of TaC at the top of the coating is significantly larger and more uniformly distributed compared to the bottom and middle regions, while the amount of Fe_7_Ta_3_ is decreasing or even disappearing.

## 4. Discussion

### 4.1. Crack Formation Mechanism Analysis

**Figure 13:**
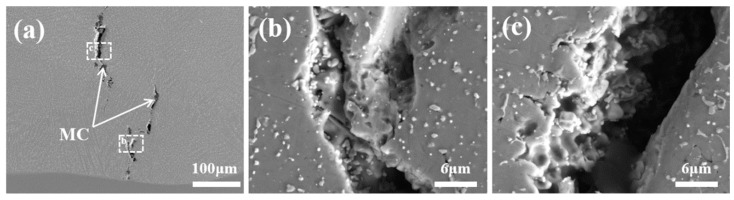
(**a**) Microscopic morphology of the crack (1100 W, 8 mm/s, 6 g/min, 41%); (**b**,**c**) localized magnification of selected regions of (**a**).

Figure 13a depicts the macroscopic morphology of the longitudinal crack. It is characterized by a long and wide crack with a zigzag pattern, a large gap between the cracked surfaces, tear marks in the cracked region, and coarse MC-type carbide particles distributed around the crack. Based on these observations, it was initially hypothesized that the longitudinal crack was thermal cracking [30,31,32]. The crack morphology was characterized to further determine the crack type. Figure 13b,c show high-magnification SE images. The fracture shows smooth and rounded dendritic features, which proves that the tensile fracture of the liquid film causes the crack. Additionally, the alloy does not have low-melting-point eutectic phases, so the type of the crack is determined to be a solidification crack under the category of hot cracking [33,34].

**Figure 14:**
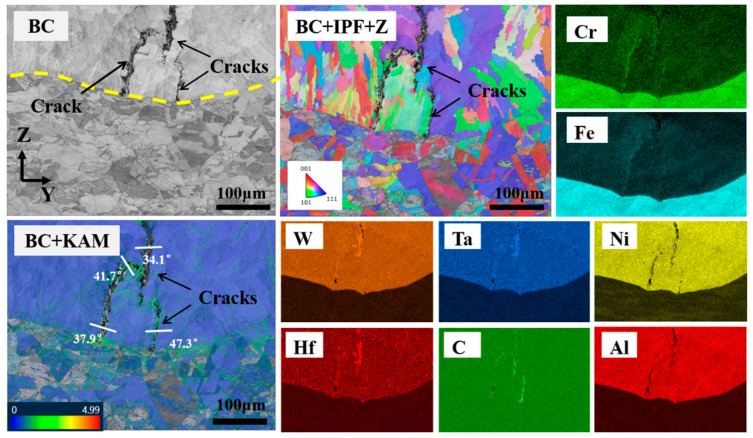
EBSD results for the crack initiation regions (1100 W, 8 mm/s, 6 g/min, 41%).

Figure 14 shows the EBSD results of the crack initiation regions. The BC and BC + IPF + Z diagrams indicate that the crack is located at the grain boundary. The columnar crystals on either side of the crack exhibit divergent growth, with a clear orientation difference. The BC + KAM diagrams illustrate the distribution of residual stress around the crack. The presence of a significant stress concentration in the vicinity of the crack and its surrounding area indicates a close correlation between the formation of the crack and the distribution of residual stress. Consequently, it can be hypothesized that the distribution of residual stress also influences the formation of the crack. The elemental distribution in the region around the crack is characterized to determine whether the crack formation is related to other factors. As shown in Figure 14, it is evident that elements such as C, Ta, Hf, W, Fe, Cr, etc., are enriched in the crack-budding position, whereas elements such as Ni and Al at the corresponding positions are significantly depleted. This further substantiates the presence of a considerable number of MC-type carbides and Fe_7_Ta_3_ phases in the crack-budding region. The presence of MC carbides and Fe_7_Ta_3_ phases indicates that both have a role in the formation of solidification cracks. It is generally believed that the effect of high-melting-point carbides on hot cracking can be divided into two categories [35,36]: (1) impeding the flow of the liquid phase, resulting in inadequate feeding and contraction within the inter-dendritic channels, while simultaneously facilitating the formation of a liquid film; and (2) weakening the bonding strength between the matrix materials. Fe_7_Ta_3_, a high-melting-point brittle and hard phase, plays a similar role to carbides.

**Figure 15:**
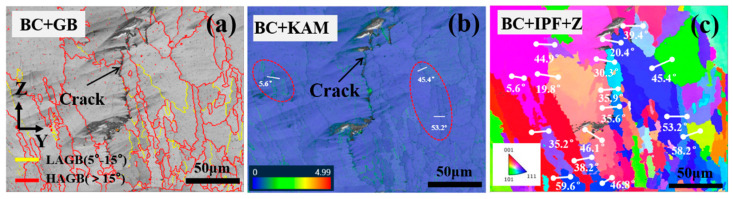
Stress distribution and orientation difference angles near the crack (1100 W, 8 mm/s, 6 g/min, 41%): (**a**) the proportion of high-angle grain boundary (HAGB) and low-angle grain boundary (LAGB); (**b**) KAM stress distribution diagram; (**c**) distribution of crystal orientation difference angles.

It is well known that the grain boundary angle is a significant factor influencing the susceptibility of alloys to cracking during LC [37,38]. The crack sensitivity has been reported to increase with the grain boundary orientation angle increasing [39,40]. A randomly selected crack initiation was characterized by EBSD to determine the relationship between the grain boundary orientation angle and cracks. The distribution of grain boundaries near the crack source is shown in Figure 15a. This figure illustrates that cracks sprout at large-angle grain boundaries, while no cracks are formed at small-angle grain boundaries. The orientation difference between grains on both sides of the crack was analyzed to obtain a quantitative value for the grain orientation difference on both sides of the crack. The selected positions and grain boundary angles are shown in Figure 15c. The analysis results indicate that the range of grain boundary angles between grains on both sides of the crack is between 25° and 45°. This is consistent with the findings of Guo et al. The reason for this is mainly that at these grain boundaries, the energy is high and the liquid film can be stabilized. Accordingly, the grain boundaries between 25° and 45° are defined as crack-sensitive, high-angle grain boundaries (S-HAGBs) [41]. In conjunction with the corresponding KAM diagram (Figure 15b) in this region, it can be observed that there is also a significant stress concentration at the crack initiation. However, it is noteworthy that there is no significant stress concentration and no crack at the 53.2° and 45.4° large-angle grain boundaries, and still no crack is formed at the small-angle grain boundary at 5.6°, although the stress concentration is considerable. It can be surmised that solidification cracks are caused by the combined effect of the grain boundary angle and residual stress, and only when the S-HAGB formed at the end of solidification has a high level of residual tensile stress will it lead to crack initiation and expansion.

**Figure 16:**
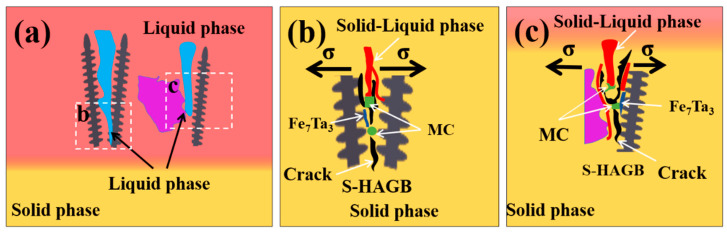
Schematic diagram of solidification crack formation mechanism: (**a**) a long solid-liquid channel is formed at the grain boundary; (**b**) the solidification cracking occurs along the S-HAGB (columnar and columnar crystals) under the action of tensile stresses; (**c**) the solidification cracking occurs along the S-HAGB (equiaxial and columnar crystals) under the action of tensile stresses.

In conjunction with the above analysis, a schematic diagram (Figure 16) was drawn to illustrate the solidification cracking mechanism of the K688 coating. The formation mechanism can be described as follows: The high-temperature gradient and high cooling rate during the laser cladding process result in the growth of grains at the bottom of the coating in the form of cellular or columnar dendritic crystals. Concurrently, the enrichment of Fe, Ta, Hf, and C at the grain boundaries promotes the formation of the high-melting-point precipitation phase and liquid film [42,43,44,45], which in turn leads to the formation of a very long solid–liquid, two-phase channel between the two grains [46] (Figure 16a). Upon solidification of the molten pool into the terminal stage, the liquid phase within the channel undergoes solidification and shrinkage. However, the high-melting-point carbides, the Laves phases, and coarse dendritic crystals at the S-HAGB impede the flow of the liquid phase, analogous to the “pegging effect” that inhibits the complementary contraction of the liquid phase at the distal end. This results in an insufficient complementary contraction of the liquid phase in the channels between the dendrites, weakening the bonding strength between the matrices. Finally, solidification cracking occurs along the S-HAGB under the action of tensile stresses (σ) (Figure 16b,c).

### 4.2. Crack Inhibition and Inhibition Cause Analysis

**Figure 17:**
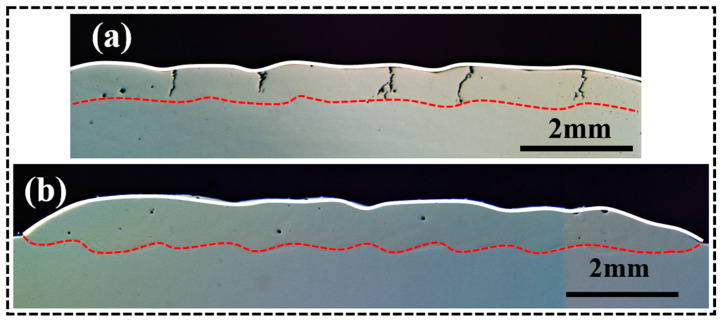
Cross-sectional (YZ) microscopic morphologies of the coatings with different laser beam patterns: (**a**) Gaussian laser beam (1100 W, 8 mm/s, 6 g/min, 41%); (**b**) flat-top laser beam (700 W, 7 mm/s, 6 g/min, 60%).

The formation mechanism of solidification cracks proposed in this paper indicates that high residual stress, coarse carbides, the Fe_7_Ta_3_ phase, and the formation of S-HAGB are the primary causes of coating cracking. Additionally, previous studies have demonstrated that, compared to the Gaussian beam, a flat-top beam with a more uniform energy distribution is beneficial for suppressing defect formation and expanding the process window of laser machining [47,48]. Therefore, based on the research of previous workers along with this paper, we propose the use of a flat-top laser beam for laser cladding. Through orthogonal experiments, we conclude that crack formation can be significantly inhibited when the laser power is 700 W, the scanning speed is 7 mm/s, the powder feeding rate is 6 g/min, and the overlap ratio is 60%. The cross-sectional microscopic morphology of the coating is shown in Figure 17. When compared with the Gaussian laser cladding coating (Figure 17a), there is no obvious crack in the coating prepared by the flat-top laser cladding, except for a few small holes (Figure 17b).

**Figure 18:**
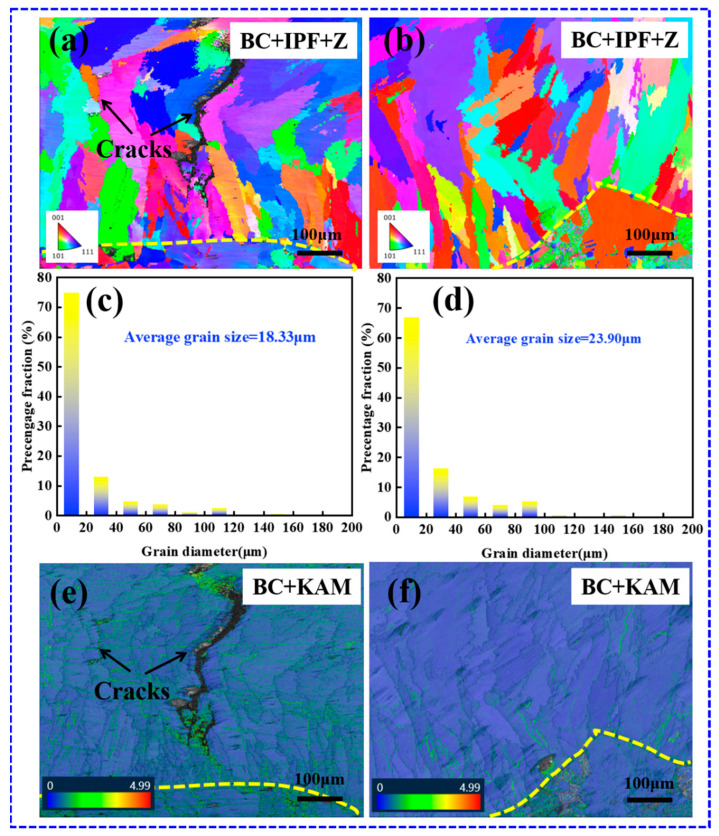
IPF map, grain diameter, and KAM map of the coatings with different laser beam patterns: (**a**,**c**,**e**) Gaussian laser beam (1100 W, 8 mm/s, 6 g/min, 41%); (**b**,**d**,**f**) flat-top laser beam (700 W, 7 mm/s, 6 g/min, 60%).

The grain morphology, diameter, and stress distribution for two styles of coatings with different laser beam patterns were analyzed to elucidate the rationale behind crack suppression, as shown in Figure 18. The morphology of the grains is shown in Figure 18a,b. Both coatings consist of anisotropic columnar and equiaxed crystals. The data on grain size in the coatings, as shown in Figure 18c,d, revealed that the average grain sizes of the Gaussian laser cladding coating and the flat-top cladding coating are 18.33 and 23.90 µm, respectively. The average grain size of the flat-top cladding coating is larger. This result is sufficient to demonstrate that the molten pools formed by the flat-top laser beam have a lower cooling rate [49]. In comparison to the Gaussian laser cladding coating, the flat-top cladding coating exhibits a markedly reduced stress concentration (Figure 18e,f), which can be attributed to the more uniform energy distribution of the flat-top laser beam, which reduces the cooling rate and temperature gradient of the molten pool formed by its cladding [50]. Moreover, the laser power and scanning speed are reduced when using the flat-top beam for cladding, which also helps to reduce the temperature gradient in the molten pool, thus alleviating the stress concentration in the coating.

**Figure 19:**
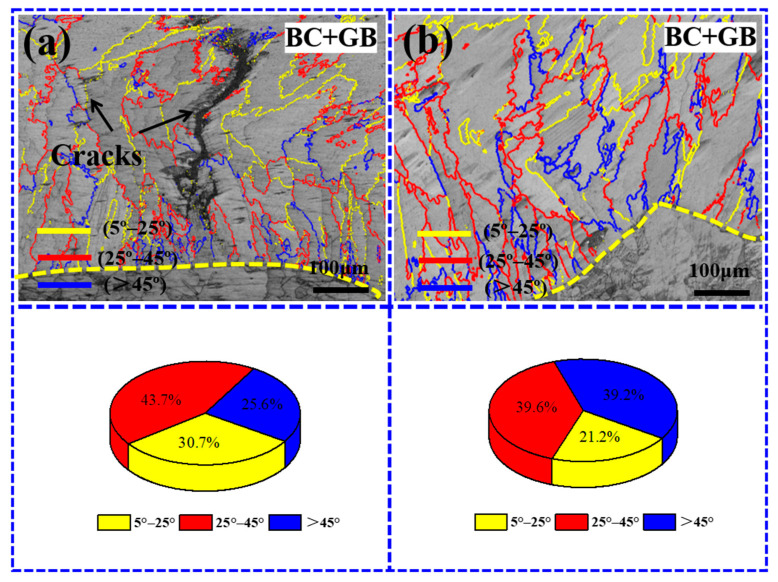
Grain boundary distributions and proportions of the coating with different laser beam patterns: (**a**) Gaussian laser beam (1100 W, 8 mm/s, 6 g/min, 41%); (**b**) flat-top laser beam (700 W, 7 mm/s, 6 g/min, 60%).

The previous studies have indicated that the grain boundary angle of the S-HAGB is 25° to 45°. Accordingly, the grain boundary angle was divided into three intervals, namely, 5° to 25°, 25° to 45°, and >45°, based on the range of the S-HAGB. The grain boundary proportions of the aforementioned three segments were subsequently subjected to statistical analysis. Figure 19a,b show the distribution and statistical results. The S-HAGB proportions of the Gaussian laser cladding coating and the flat-top cladding coating are 43.7% and 39.6%, respectively. Furthermore, the proportion of S-HAGBs in the flat-top cladding coating is reduced by approximately 10% compared to that of the Gaussian laser cladding coating. For the flat-top laser cladding coating, the possible reason for the decrease in the S-HAGB proportion is that a molten pool formed by the flat-top laser beam is more stable, which is conducive to alleviating the violence of the fluid flow in the molten pool [51,52].

In conclusion, the ultra-dramatic suppression of coating cracking achieved using a flat-top laser beam can be attributed to two main factors: the lower stress level and smaller S-HAGB formation tendency. Therefore, the utilization of flat-top laser beam cladding is highly effective in mitigating the occurrence of solidification cracks within the coating.

## 5. Conclusions

This study investigated the influence of process parameters on the microstructure and dilution ratio of the K688 coating, and its phase composition was analyzed. This analysis focused on studying the formation mechanism of hot cracks in this alloy. Based on the cracking mechanism, we almost eliminated the hot cracks in the coating by regulating the laser energy distribution. At the same time, the reasons for the elimination were discussed. The results of the study indicate the following:

- The process parameters significantly affect the cracking tendency and elemental dilution of the cladding coatings. Using medium–low power, a fixed scanning speed, a higher powder feeding rate, and a larger overlap ratio are favorable in reducing the coating cracking susceptibility and matrix element dilution. Consequently, the quality of laser cladding coatings has been improved.
- K688 powder is mainly composed of γ-Co and MC-type carbides, while in the K688 coating, in addition to γ-Co and MC-type carbides, a small amount of Fe_7_Ta_3_ phase also appeared, with the generation of the new phase due to the diffusion of matrix Fe element.
- The crack fracture of the K688 coating exhibits a developed dendrite morphology and evident elemental segregation, indicating its classification as a solidification crack under the category of hot cracking. Most cracks are along high-angle grain boundaries, with an angle ranging from 25° to 45°. The occurrence of solidification cracking is attributed to the stable presence of a liquid film at the crack-sensitive, high-angle grain boundaries (S-HAGBs), the stress concentration, the formation of the Fe_7_Ta_3_ phase, and the formation of coarse carbides.
- Compared to the Gaussian beam, the flat-top beam demonstrates a reduced cooling rate and temperature gradient, effectively mitigating tensile thermal stress within the coating. Simultaneously, this specific beam may generate a more stable melt pool, aiding the mitigating turbulent fluid flow and restraining the occurrence of S-HAGB. Therefore, crack formation can be significantly inhibited with optimized parameters, such as a flat-top beam with a laser power of 700 W, scanning speed of 7 mm/s, powder feeding rate of 6 g/min, and overlap ratio of 60%.

## Figures and Tables

**Table 1:** Chemical composition of K688 cobalt-based alloy powder (wt.%).

Co	Ni	W	Ta	Cr	Al	Hf	C	Zr	B
Bal.	25.0	15.0	8.0	5.0	4.0	1.5	0.15	0.06	0.024

**Table 2:** Single-factor experimental parameters of laser cladding.

Laser Power	Scanning Speed	Powder Feeding Rate
600–1600 W	6–13 mm/s	4–10 g/min

## Data Availability

The raw/processed data required to reproduce these findings cannot be shared at this time as the data also form part of an ongoing study.
